# The titin N2B and N2A regions: biomechanical and metabolic signaling hubs in cross-striated muscles

**DOI:** 10.1007/s12551-021-00836-3

**Published:** 2021-09-09

**Authors:** Robbert J. van der Pijl, Andrea A. Domenighetti, Farah Sheikh, Elisabeth Ehler, Coen A. C. Ottenheijm, Stephan Lange

**Affiliations:** 1 Department of Cellular and Molecular Medicine, University of Arizona, Tucson, AZ USA; 2 Shirley Ryan AbilityLab, Chicago, IL USA; 3 Department of Physical Medicine and Rehabilitation, Northwestern University, Chicago, IL USA; 4 Division of Cardiology, School of Medicine, UC San Diego, La Jolla, CA USA; 5 Randall Centre for Cell and Molecular Biophysics, School of Cardiovascular Medicine and Sciences, King’s College London, London, UK; 6 Department of Physiology, Amsterdam University Medical Centers, Amsterdam, The Netherlands; 7 Department of Molecular and Clinical Medicine, University of Gothenburg, Gothenburg, Sweden

**Keywords:** Muscle, Titin, Sarcomere, Signaling, Mechanosensor, Muscle mechanics

## Abstract

Muscle specific signaling has been shown to originate from myofilaments and their associated cellular structures, including the sarcomeres, costameres or the cardiac intercalated disc. Two signaling hubs that play important biomechanical roles for cardiac and/or skeletal muscle physiology are the N2B and N2A regions in the giant protein titin. Prominent proteins associated with these regions in titin are chaperones Hsp90 and αB-crystallin, members of the four-and-a-half LIM (FHL) and muscle ankyrin repeat protein (Ankrd) families, as well as thin filament-associated proteins, such as myopalladin. This review highlights biological roles and properties of the titin N2B and N2A regions in health and disease. Special emphasis is placed on functions of Ankrd and FHL proteins as mechanosensors that modulate muscle-specific signaling and muscle growth. This region of the sarcomere also emerged as a hotspot for the modulation of passive muscle mechanics through altered titin phosphorylation and splicing, as well as tethering mechanisms that link titin to the thin filament system.

## Introduction

**Fig. 1:**
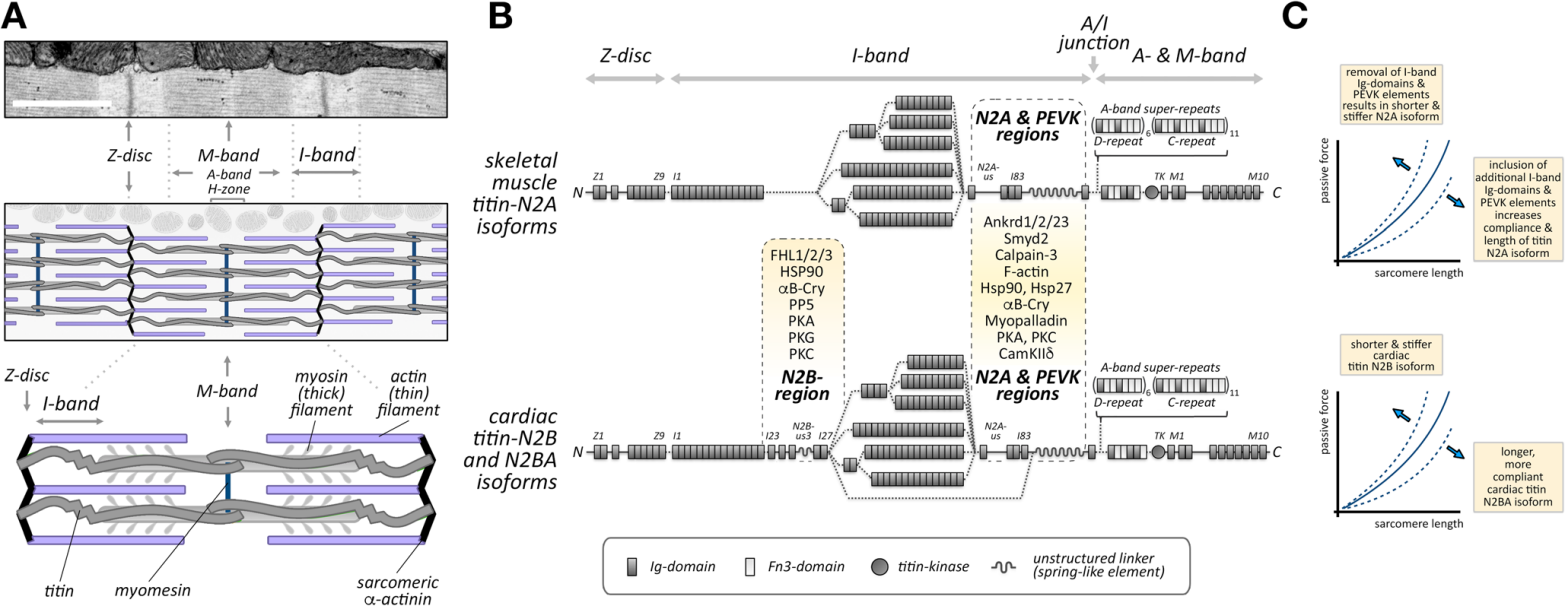
**A** Electron micrograph of cardiac muscle (top panel) identifying regions of the sarcomere (I-band and A-band) as well as structural cross-linkers (Z-disc and M-band) also presented in the schematic overview (middle panel) and in the sarcomeric unit (bottom panel). Identity of filament systems (titin, myosin and actin filament) and important crosslinker proteins (sarcomeric α-actinin and myomesin) are shown. **B** Schematics of skeletal and cardiac titin N2A (top panel) and N2BA splice isoforms (bottom panel), respectively (not to scale). The N2A region, present in both cardiac and skeletal titin, and the cardiac specific N2B region are highlighted, as are known binding partners. **C** Changes to passive force development by titin depending on I-band splicing of skeletal muscle titin N2A isoform (top panel) or cardiac N2B and N2BA isoforms (bottom panel)

The myofilaments of cross-striated muscle cells provide mechanical power for the contraction of the heart or the movement of the skeletal muscles. The machinery that drives the power development also serves additional functions: as a mechanosensory unit that provides constant feedback on the current power requirements, as a signaling node that integrates the input of muscle specific components and sensors with common cellular signaling pathways to modulate the muscle gene program and as finely tuned regulators of not only active but also passive force development and tension. Many of these functions emanate from and are mediated by three filament systems: actin, myosin and titin filaments, as well as an intricate system of accessory proteins and cellular structures, such as the intercalated discs (specialized cell-cell contacts in cardiac muscle cells) or costameres (structures that connect the sarcomere to the muscle cell membrane and extracellular matrix). Together, the three filament systems make up the sarcomere, the smallest contractile unit in myofilaments (reviewed in Gautel and Djinovic-Carugo (2016) and Henderson et al. (2017)). Several regions within the sarcomere can be discerned from microscopic images, such as the A-band, which is formed by the myosin (thick) filaments and appears as electron-dense regions within electron micrographs (Figure 1A). Interspersed between the A-bands are the I-bands, which contain the actin (thin) filaments and part of the elastic filaments formed by the giant protein titin. Crosslinkers in the Z-disc and M-band regions provide structural stability and link the three filament systems together (Lange et al. 2020; Luther 2009).

Over the last decades, the giant protein titin has emerged as a master regulator for muscle development, function, signaling and maintenance and as a modulator of passive muscle mechanics (Herzog 2018; Kotter et al. 2014a; Kruger and Kotter 2016; Linke 2008). Titin is the largest protein in the human body and spans ~1 μm from the sarcomeric Z-disc to the M-band. The protein is expressed in three main full-length isoforms in adults: shorter, stiffer N2B and longer, more compliant N2BA isoforms in the heart, as well as the N2A isoform in skeletal muscles (Figure 1B). Additional splice isoforms that are reminiscent of the N2BA isoform can be found during embryonic and postnatal development of the heart (Lahmers et al. 2004; Opitz et al. 2004) and in skeletal muscles (Li et al. 2012). Smaller titin isoforms like Cronos or Novex-3 that only incorporate part of the full titin sequence have also been identified (Kellermayer et al. 2017; Labeit et al. 2006; Zaunbrecher et al. 2019). However, their role is much less characterized and may extend beyond cross-striated muscles.

Much of titin splicing happens in the I-band region of the protein, either by including or removing additional Ig-domains that change the overall contour length of the protein, or by altering the length of the elastic PEVK element, named after its proline (P), glutamate (E), valine (V) and lysine (K) rich content, which modulates titin compliance (Figure 1C) (Bang et al. 2001a; Gautel et al. 1996; Linke et al. 1999). The I-band portion of titin also contains two major signaling hubs, the N2A and N2Bregions, which in addition to tuning cellular signaling pathways also influence passive muscle mechanics.

## Signaling and biomechanical functions of titin’s N2A region

**Fig. 2:**
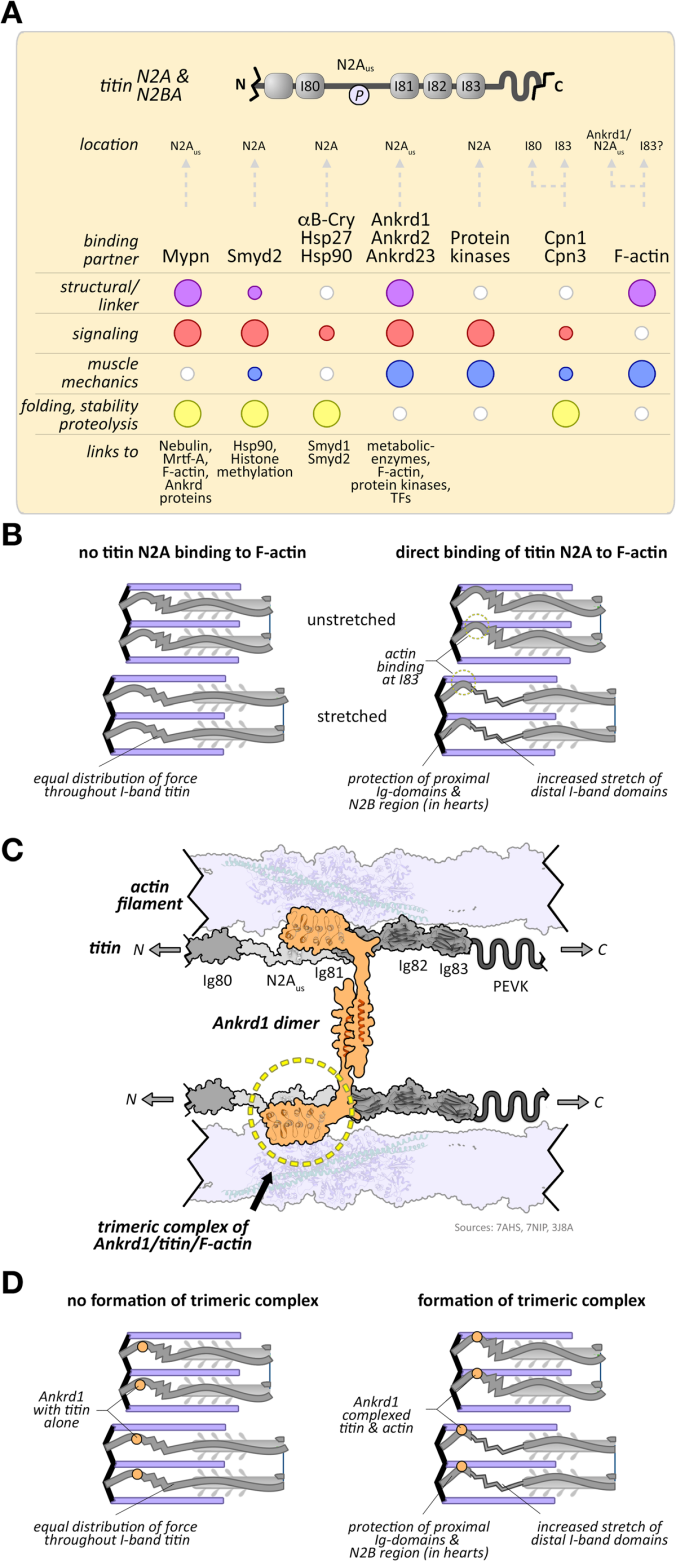
**A** Schematic representation of titin N2A region domain structure (top), encompassing Ig-domain 80 (I80) to Ig-domain 83 (I83). The titin N2A_us_ linker and PEVK element are shown (P = phosphorylation). Location of binding site(s) within titin for each interaction partner, their roles and linkage to other proteins are also depicted (bottom panel; TFs = transcription factors). The described functional roles for each protein are visualized by circles colorized for each category, with small white circles indicating lack of evidence, and larger circles representing strong experimental support. **B** Influence of direct titin I83 binding to F-actin (right panel) on I-band titin in unstretched and stretched muscle. Linkage of titin I83 to actin leads to increased stretch of distal I-band domains and the PEVK element, protecting proximal Ig-domains from unfolding and preventing stretch of the N2B region (in cardiac titin isoforms). **C** Schematic representation of the Ankrd-dimer linking to adjacent titin filaments at the N2A_us_ region, and of the Ankrd1/titin/F-actin trimeric complex that ‘locks’ titin to the actin filament. Structures shown in the representation are adapted from RCSB accession 7AHS (Stronczek et al. 2021), 7NIP (Zhou et al. 2021) and 3J8A (von der Ecken et al. 2015). **D** Effect of Ankrd binding to I-band titin alone (left panel) or in complex with F-actin (right panel) in unstretched (top panel) and stretched muscle (bottom panel). Formation of the trimeric Ankrd1/titin/F-Actin complex leads to increased stretch of distal I-band domains and PEVK element, protecting proximal Ig-domains from unfolding and preventing stretch of the N2B region (in cardiac titin isoforms)

The N2A region of titin is found in both cardiac and skeletal muscles (Figures 1B and 2A). This region of titin is located just N-terminal of titin’s large, disordered PEVK region, named for its high percentage of proline (P), glutamate (E), valine (V) and lysine (K) residues. Three Ig-domains, I81–I83, separate the N2A and PEVK regions (Bang et al. 2001a). While long thought of as another flexible and largely disordered linker surrounded by Ig-domains, it recently emerged that the N2A region contains more structural elements than previously anticipated. Biochemical and biophysical analyses of a purified N2A fragment indicated that the N2A unique sequence (N2A_us_) contains a core of interacting α-helices with unusual structural and thermal stability surrounded by disordered elements. The N2A_us_ region assumes an overall elongated monomeric conformation (Tiffany et al. 2017; Zhou et al. 2016). Further structural analysis of the extended N2A element suggested additional stabilizing interactions with the C-terminal Ig-domain 81 (I81) through its uncommon BC-loop (Zhou et al. 2016). Ig-domains I81–I83 in titin form an atypical set of immunoglobulin-like domains, which are distinct from other Ig-domains in titin (Stronczek et al. 2021). Atomic force microscopy studies revealed that the N2A region acts as an entropic spring element, whose unfolding force/persistence length but not contour length is actively modulated by one of its interaction partners: Ankrd1 (Lanzicher et al. 2020).

Several phosphorylation sites have been mapped to the N2A_us_ linker and the surrounding Ig-domains, which are targets of cAMP-dependent protein kinase (PKA) (Adams et al. 2019; Lanzicher et al. 2020) and cGMP-dependent protein kinase (PKG) (Kruger et al. 2009). One of the phosphorylation sites, S9895 in NP_001254479.2, is located at the interface between N2A_us_ and I81 (Adams et al. 2019; Lanzicher et al. 2020). Despite the location, this phosphorylation site does not affect the biomechanical properties of this area in titin; instead, S9895 phosphorylation may modulate binding of titin-associated proteins and strain-dependent signaling (Lanzicher et al. 2020). PKA-mediated phosphorylation of the N2A_us_ S9895 can be quenched by binding to Ankrd1 (Lanzicher et al. 2020; Lun et al. 2014), suggesting blockage of kinase access to titin.

The N2A region has been reported to bind calcium, which might facilitate its binding to actin (Dutta et al. 2018). However, this is a debated subject with experimental support for both, calcium-mediated actin binding and no direct interaction to the thin filament. Although contested, binding of N2A to the thin filament would have a dramatic impact on titin compliance and forces acting on I-band domains and elements, as proximal titin domains closer to the Z-disc may be protected from stretch-induced damage, while distal domains up to the A/I-junction (including the flexible PEVK element) will experience enhanced stretch (Figure 2B) (Nishikawa et al. 2020a). This model was proposed by Krysta Powers and Gudrun Schappacher-Tilp et al. and further refined by Kiisa Nishikawa et al., who demonstrated thin filament binding to I83 (Dutta et al. 2018; Kelly et al. 2021; Kelly et al. 2020; Powers et al. 2016; Powers et al. 2014; Schappacher-Tilp et al. 2015). However, results demonstrating or disputing association of N2A with actin appear to depend on the construct studied and/or presence/absence of calcium. Constructs spanning I80–I83 (Dutta et al. 2018; Nishikawa et al. 2020a) and I80–I81 (van der Pijl et al. 2021) have been shown to interact with actin in a calcium-dependent manner, whereas constructs encompassing just N2A_us_, N2A_us_-I81, I80–I82 and I81–I83 do not appear to interact with actin, independent of calcium (Linke et al. 1997; Stronczek et al. 2021; Zhou et al. 2021). Additionally, it remains unclear if this mechanism of I83 binding to the thin filament is present in vivo, as actin interaction with proteins may sometimes be non-specific due to the high abundance of the protein. The unspecific binding of abundant muscle proteins, such as actin or myosin heavy chain is a point of concern for many studies. It is also important to consider that folding of proteins heterologously expressed in bacteria can be heterogeneous and lacks many of the posttranslational modifications found in eukaryotes, giving rise to subpopulations of proteins with improper functions and binding properties that might confound results (Palomares et al. 2004; van der Lee et al. 2014). Thus, studies with recombinant titin fragments are challenging, and the interpretation of the results requires careful considerations.

Adding to this already complex problem of titin N2A association with the thin filaments is the recent finding that Ankrd1 crosslinks titin N2A to F-actin (Figure 2C) (van der Pijl et al. 2021; Zhou et al. 2021). This trimeric protein complex would result in similar effects on passive muscle mechanics as the direct binding of N2A-I83 to F-actin: protecting proximal titin domains in favor of imposing increased stretch on the PEVK element and Ig-domains (including I83) up to the A/I junction (Figure 2D). Data from Ankrd knockout models that show longer sarcomere lengths, more compliant muscles and greater susceptibility for injuries following eccentric exercise are supportive of the biological relevance for the trimeric Ankrd/titin-N2A/F-actin complex (van der Pijl et al. 2021; Zhou et al. 2021). Further analysis of mice with muscular dystrophy with myositis (MDM mice) that have a naturally occurring mutation in I83 (Garvey et al. 2002) may provide additional insight into the biological relevance of the Ankrd1-mediated actin locking mechanism, specifically when crossed with Ankrd knockout mice. Published data demonstrate increased expression and localization of Ankrd1 (and Ankrd2) to the sarcomeres in MDM mouse muscles (Mohamed et al. 2013; Witt et al. 2004).

An intriguing side effect of both, formation of either the trimeric Ankrd/titin-N2A/F-actin complex or the direct linkage of I83 to F-actin, is the possibility that biomechanical signaling at the N2B region in cardiac titin may be affected. Similar to the proximal Ig-domains, the N2B region is also located towards titin’s N-terminus relative to the Ankrd binding site or I83. Hence, cross-linkage of titin to the thin filament at the N2A region should also protect the N2B region from stretch, potentially modifying biomechanical signaling and passive force exerted from this part of titin. However, further experiments that interrogate the potential biomechanical cross-talk between the N2B and N2A regions in titin are needed.

## Titin N2A binding to modifying enzymes, chaperones and proteases

The N2A signalosome is an expanding signaling hub for proteins involved in stability, proteolysis, control over muscle growth and muscle mechanics (Figure 2A). Various chaperone proteins bind along the extensible I-band region of titin, including the N2A region. Small heat shock proteins, Hsp27 (Hspb1) and αB-crystallin increase titin-based passive tension and have been linked to various myopathies (Kotter et al. 2014b; Unger et al. 2017). The methyltransferases SET and MYND domain containing 1 and 2 (Smyd1 and Smyd2, respectively) are highly expressed in cross-striated muscles, and Smyd2 has been shown to bind to the N2A region (Donlin et al. 2012; Voelkel et al. 2013). Both enzymes methylate histones and non-histone proteins, such as p53 or Hsp90. Complex formation between titin, Smyd2 and Hsp90 was demonstrated to be dependent on Hsp90 methylation (Donlin et al. 2012) and is thought to protect titin’s I-band region from oxidative damage and to maintain sarcomere organization (Donlin et al. 2012; Munkanatta Godage et al. 2018; Voelkel et al. 2013). Loss of function studies suggest that Smyd1 (also called Bop) is required for cardiomyogenesis and sarcomere assembly in skeletal and cardiac muscles (Gottlieb et al. 2002). This role for Smyd1 is evolutionary conserved, as muscle formation and function were also affected in morpholino-targeted *Xenopus laevis* animals (Kawamura et al. 2008). In contrast to critical roles for Smyd1 in cross-striated muscles, Smyd2 is dispensable for heart development (Diehl et al. 2010). However, Smyd2 activity was shown to be important for titin stability and skeletal muscle function, as its deficiency in zebrafish resulted in severely impaired mobility and contracted tails (Donlin et al. 2012).

The Ca^2+-^dependent protease family members calpain 1 and calpain 3 (also called p94) bind both to titin. Calpain 1 binds near the Z-disc at I4 (Coulis et al. 2008) and the N2A region (Raynaud et al. 2005), with its binding affinity regulated by calcium. At high calcium levels, calpain 1 is bound to titin in the inactive form where it is thought to form a reservoir until it is activated, possibly in response to mechanosensing responses or damage of titin domains. The skeletal muscle specific calpain 3 binds to titin N2A through its is2 domain, and there are indications that the binding is possibly mediated by phosphorylation at S629 (Ojima et al. 2014). The binding to titin is also thought to maintain calpain 3 in its inactive form. Besides interacting with I82–I83 in the N2A region (Hayashi et al. 2008; Ojima et al. 2007), calpain 3 also associates with the I80-N2A_us_ and the PEVK regions (Hayashi et al. 2008), as well as with M-band titin (Sorimachi et al. 1996). Calpain 3 has been shown to translocate from the M-band to the N2A region in a load-dependent manner (Ojima et al. 2010; Ojima et al. 2007). This sensing response in sarcomeres is thought to trigger calpain 3 autolytic activity and initiate downstream functions (Nishikawa et al. 2020b). Mutations in the calpain 3 binding sites in/close to N2A can result in muscular dystrophy, such as seen in the MDM mouse (Garvey et al. 2002). In the N2A signalosome, calpain 3 also interacts/proteolytically cleaves members of the muscle ankyrin repeat (Ankrd) protein family (Laure et al. 2010; Ojima et al. 2010).

## Muscle ankyrin repeat proteins (Marps)—masters of titin-based signaling and titin-based muscle compliance

The Ankrd protein family contains three members: Ankrd1 (Marp1, Carp1, C-193), Ankrd2 (Marp2, Arrp [ankyrin repeat protein with pest motif and proline-rich region], Carp2) and Ankrd23 (Marp3, Darp [diabetes-related ankyrin repeat protein], Carp3). All family members are structurally similar and consist of an N-terminal coiled-coil domain that allows for homodimerization and heterodimerization of Ankrd1, Ankrd2 and Ankrd23 (Lun et al. 2014; Witt et al. 2005), followed by a centrally located unstructured linker that contains a nuclear localization sequence, and a series of ankyrin-repeats towards the C-terminus that facilitate binding to various proteins (Chu et al. 1995; Miller et al. 2003). Ankrd1 and Ankrd2 also contain several putative proline (P), glutamic acid (E), serine (S), threonine (T)-rich (PEST) sequences that target the proteins for degradation (Chu et al. 1995; Lun et al. 2014). All Ankrd members are involved in hypertrophic and atrophic signaling pathways in the heart, and there is a growing body of literature showing effects of these proteins also in skeletal muscle. Ankrd protein interaction with N2A was first described by Miller and coworkers (Miller et al. 2003), who showed that all members of this protein family bind to the N2A_us_ region through their respective second ankyrin repeat, and to a lesser extent with their N-terminal sequences. Subsequently, it was suggested that full-length Ankrd proteins may crosslink adjacent titin proteins (Figure 2C) by forming antiparallel homodimers or heterodimers (Lun et al. 2014), and the binding site in N2A was refined to include the adjacent I81 in addition to N2A_us_ alone (Zhou et al. 2016). Cleavage by calpain 3 was demonstrated to cut Ankrd1 and Ankrd2 in or near their coiled-coil region, forming a mechanism for disrupting Ankrd dimerization (Hayashi et al. 2008). Cleavage of Ankrd1 appears to reinforce binding of the protein to titin N2A (Laure et al. 2010). All Ankrd proteins display muscle specific expression under baseline conditions (Miller et al. 2003; Wette et al. 2017), with Ankrd1 being primarily detected in the heart and at very low levels in skeletal muscle, Ankrd2 being present mostly in slow-twitch skeletal muscle fibers and exhibiting low expression in the heart and fast-twitch fibers and Ankrd23 having low-level expression in striated muscle.

### Ankrd1 functions in cross-striated muscles

Ankrd1 is the best studied member of the Ankrd protein family. Investigations of Ankrd1 functions have been primarily focused on the heart where the protein is thought to regulate signaling and transcription (Ling et al. 2017). Ankrd1 expression is regulated through Gata4 and Nkx2.5 (Chen et al. 2012; Kuo et al. 1999; Zou et al. 1997). Once formed, Ankrd1 complexes with Gata4-Erk1/2 to induce phosphorylation of the transcription factor. This in turn stimulates the nuclear localization of Gata4, which further activates the hypertrophic gene program (Zhong et al. 2015). Other transcription factors that have been shown to utilize Ankrd1 as transcriptional co-activator or -repressor include Yb-1 (Zou et al. 1997), nucleolin (Almodovar-Garcia et al. 2014), p53 (Kojic et al. 2010) or NF-κB (Liu et al. 2015). NF-κB p65 DNA-binding activity is decreased during Ankrd1 overexpression, a mechanism that has been linked to reduced cell survival and is tied to calpain 3 activity (Laure et al. 2010; Mohamed and Boriek 2012). Calpain-3 cleaved Ankrd1 negatively modulates NF-κB p65 DNA-binding activity (Laure et al. 2010). Ankrd1 also binds to the N-terminus of myopalladin (Bang et al. 2001b), a protein that was also shown to localize to the Z-disc via its interaction with sarcomeric α-actinin (Bang et al. 2001b; Huby et al. 2014).

Loss of Ankrd1 in mice is well-tolerated, as is deletion of the other individual Ankrd family members, or a combination of all in Ankrd1/Ankrd2/Ankrd23 triple knockout mice (Bang et al. 2014; Barash et al. 2007). While most physiological and morphological parameters in cardiac and skeletal muscles were unchanged, triple Ankrd knockouts showed a subtle but noticeable increase in sarcomere length and muscle compliance. These changes were accompanied by greater muscle injury following eccentric contraction exercise, again indicative of protective functions of Ankrd proteins (Barash et al. 2007).

Ankrd1 levels are increased in many myopathy types, suggesting the use of the protein as a biomarker (Ling et al. 2017). However, it is contested if elevated cardiac Ankrd1 levels alone promote the development of cardiomyopathy, as two independent studies using transgenic Ankrd1 mice gave opposing results: one finding no overt physiological abnormality of transgenic mice, while the other showed diastolic dysfunction (by shifting the titin isoforms ratio towards the stiffer N2B version) and progressive heart failure (Piroddi et al. 2020; Song et al. 2012). However, crossbreeding Ankrd1 knockouts to muscle lim protein (MLP/Csrp3) knockout mice gave a major clue to the role of Ankrd1 in the heart. While MLP knockouts develop dilated cardiomyopathy (Arber et al. 1997), MLP/Ankrd1 double knockout mice display normal cardiac morphology and systolic function (Lange et al. 2016). Further analysis revealed that the elevated Ankrd1 levels in MLP knockouts promote the activation of protein kinase C α (PKCα), which underlies the etiology of DCM in these mice (Braz et al. 2004; Hambleton et al. 2006). Intriguingly, Ankrd1 translocated from the N2A region to the intercalated disc, a pathological hallmark that was also seen in cardiac biopsies of DCM patients (Lange et al. 2016). It remains unclear how this translocation is regulated, but data from 2D-gel electrophoresis experiments indicate a role in the posttranslational modification of Ankrd1 (Lange et al. 2016).

Many functions for Ankrd1 in skeletal muscle remain to be further explored and discovered. Similar to the heart, Ankrd1 is upregulated in skeletal muscle upon cellular stress (Buck et al. 2014; Kojic et al. 2004; Laure et al. 2009; Liu et al. 2015; van der Pijl et al. 2018; van der Pijl et al. 2020; van der Pijl et al. 2021; Witt et al. 2004), and the protein seems to play a role in atrophic signaling through NF-κB (Liu et al. 2015) and fiber type switching through p21 (Waf1/Cip1) (Laure et al. 2009).

Two other recent studies established a novel role for Ankrd1 in the regulation of muscle compliance: by crosslinking titin to the thin filaments in skeletal muscle at the level of the N2A region (Figure 2B) (van der Pijl et al. 2021; Zhou et al. 2021). Besides altered titin association to filamentous actin (Dutta et al. 2018; Kelly et al. 2021; Kulke et al. 2001; Linke et al. 2002; Nagy et al. 2004; Nishikawa et al. 2020a; Nishikawa et al. 2020b), variation of its splice isoforms and changed posttranslational modifications at the N2B_us3_ and PEVK regions (reviewed in Hidalgo and Granzier (2013) Kruger and Linke (2009) and Linke and Hamdani (2014)), this crosslinking mechanism offers yet another avenue to modulate titin-based muscle stiffness in an hitherto unprecedented fashion. The formation of the trimeric Ankrd1, titin and F-actin protein complex is mediated by the C-terminal ankyrin repeats in Ankrd1, although it is unclear how the locking mechanism is regulated and what role the Ankrd1 N-terminus plays. While Ankrd1 binding to titin is independent of a mapped PKA phosphorylation site located in the N2A_us_ region (Adams et al. 2019; Lanzicher et al. 2020), extensive posttranslational modifications of Ankrd1 and/or titin by PKA and other protein kinases may play a role in complex establishment and dissociation (Lun et al. 2014). Further experiments are needed to characterize the molecular and biomechanical properties of this complex and determine its role for the heart.

More studies are also needed to solidify the link between Ankrd1 and muscle signaling and maintenance in skeletal muscle. One possible mechanism might involve the heat-shock proteins Hsp90, Hsp27 or αB-crystallin, which have been shown to also associate with the titin N2A region (Donlin et al. 2012; Kotter et al. 2014b; Unger et al. 2017; Voelkel et al. 2013) and were linked to sarcomere stability and control over muscle growth (Huey et al. 2010; Senf et al. 2013).

### Roles of Ankrd2 for skeletal and cardiac muscles

Ankrd2 (also known as Arrp [ankyrin repeat protein with pest motif and proline-rich region]) is the more prominently expressed Ankrd protein family member in skeletal muscle (Miller et al. 2003; Wette et al. 2017). Like Ankrd1, Ankrd2 can interact with many transcription factors such as Ap-1, Mef2C or p53 (Belgrano et al. 2011; Kojic et al. 2004) (reviewed in Cenni et al. (2019)). In response to oxidative stress, Ankrd2 can be phosphorylated at S99 by Akt2, which prompts binding of Ankrd2 to NF-κB p50. This inhibits p50 in a manner similar to Inhibitor of κB (IκB) and suppresses NF-κB driven gene transcription and myogenesis (Bean et al. 2014; Cenni et al. 2011). Ankrd2 also associated with Tcap (Kojic et al. 2004) and LIM domain-binding 3 (Ldb3; Zasp; Cypher) in the Z-disc to regulate PKCα or mTOR activity (Martinelli et al. 2014; Pathak et al. 2021). Similar to Ankrd1, Ankrd2 can be cleaved by calpains 1 and 2 (Piatkov et al. 2014) and likely by calpain 3 (Cenni et al. 2019; Ojima et al. 2010), which removes the coiled-coil domain, possibly resulting in a more stable Ankrd2 fragment (Piatkov et al. 2014). Also similar to Ankrd1, Ankrd2 binds to titin’s N2A region (Lun et al. 2014; Miller et al. 2003), suggesting redundant or similar roles for Ankrd family members. Indeed, loss of Ankrd2 in MLP knockout mice also delayed DCM development (Lange et al. 2016). While this ‘rescue’ of the MLP knockout phenotype is less complete compared to MLP/Ankrd1 double knockouts, it substantiates functional redundancy between Ankrd family members in vivo. However, it remains to be seen if the formation of the trimeric complex with titin and filamentous actin, and the subsequent regulation of muscle compliance demonstrated for Ankrd1 extends to the other Ankrd family members.

### Ankrd23—the mysterious third member of the muscle ankyrin repeat protein family

Ankrd23 (also known as Darp [diabetes-related ankyrin repeat protein]) is the least understood member of the Ankrd protein family. Several studies indicated that this Ankrd family member might be involved in metabolic signaling (Ikeda et al. 2003; Shimoda et al. 2015) and the regulation of skeletal muscle differentiation (Wang et al. 2017). Ankrd23 interacts with both titin N2A and myopalladin, and displays similar to other Ankrd family members stretch-induced redistribution from its sarcomeric localization, albeit preferentially to the intercalated discs (Miller et al. 2003).

## Myopalladin, a crucial protein for muscle health

Besides binding to the N2A_us_ region of titin, Myopalladin is also found at the Z-disc, where it was shown to interact with muscle specific α-actinin isoforms Actn2 and Actn3 (Bang et al. 2001b) as well as nebulin (Ma and Wang 2002; Witt et al. 2006). The role of Myopalladin for the maintenance of Z-disc structure is underscored by the loss-of-function phenotype, which displayed Z-disc damage with exercise, and Z-disc widening with age. Knockout mice were smaller, had more atrophied muscles (measured as reduced cross-sectional area) and displayed decreased exercise capacity (Filomena et al. 2020). Mechanistically, deletion of Myopalladin resulted in reduced serum response factor (Srf) activity that was caused by loss of Myopalladin interaction with Myocardin-related transcription factor A (Mrtf-A). The skeletal muscle phenotype could be rescued in Myopalladin knockout myoblasts by transduction of a constitutively active Srf, which promoted muscle growth and increased myotube widths.

Binding of Myopalladin to Ankrd1 is mediated by the Myopalladin N-terminus. Overexpression of the N-terminus in cardiac myocytes resulted in marked disruption of Z-disc and M-band architecture as well as thin filament disorganization (Bang et al. 2001b). Underscoring the importance for Myopalladin in cross-striated muscles is the fact that a number of pathological mutations are associated with several types of skeletal and cardiac myopathies, including hypertrophic, dilated or restrictive cardiomyopathy as well as nemaline myopathy (reviewed in Wadmore et al. (2021)).

## Titin’s N2B region as a metabolic and cellular signaling hub that modulates passive titin mechanics

**Fig. 3.**
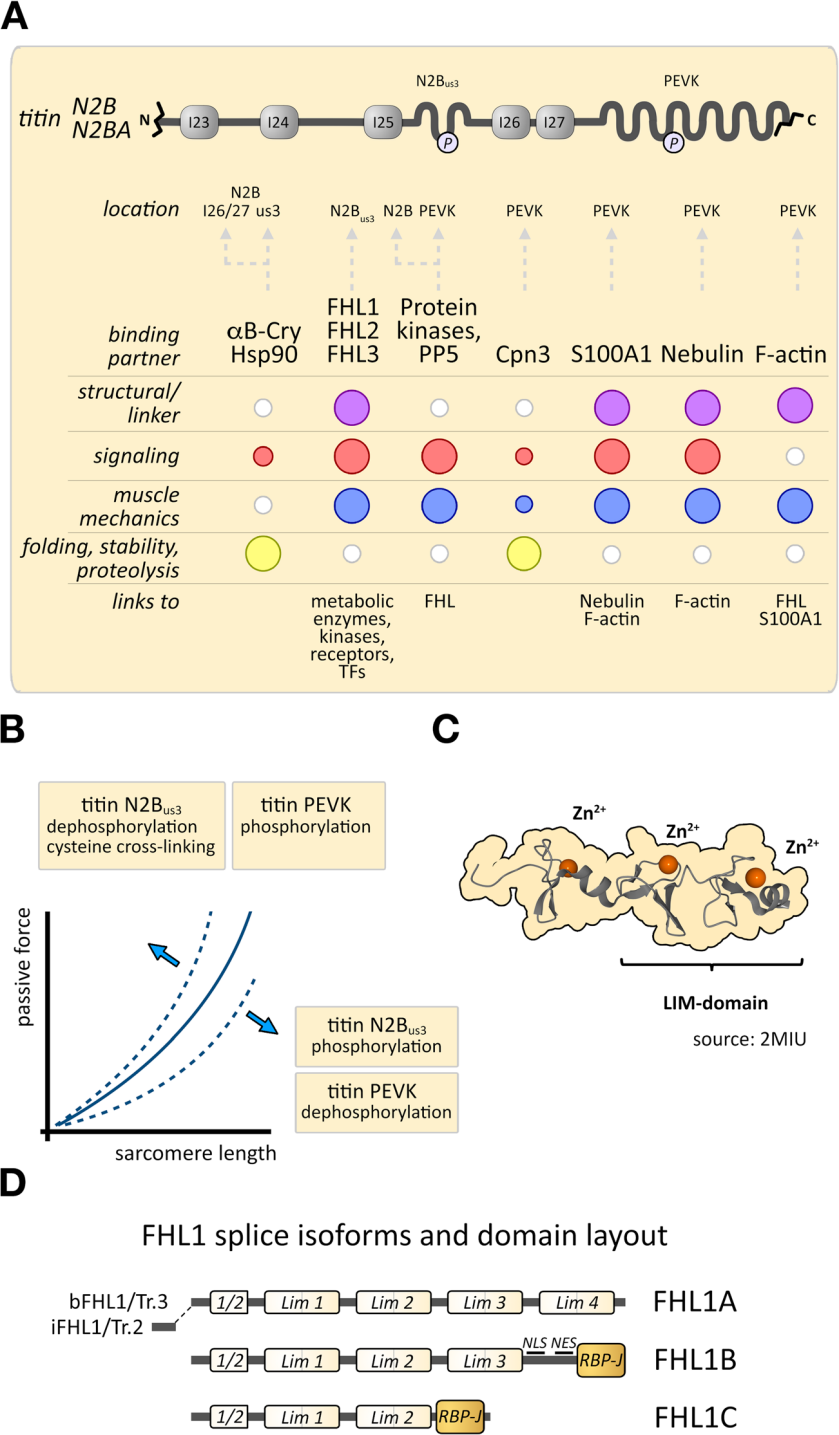
**A** Schematics of the titin N2B region, spanning Ig-domains Ig23 (I23) to Ig27 (I27), with positions of the N2B_us3_ region and PEVK element highlighted. Location of binding site(s) within titin for each interaction partner, their roles and linkage to other proteins are also depicted (bottom panel; TFs = transcription factors). The described functional roles for each protein are visualized by circles colorized for each category, with small white circles indicating lack of evidence, and larger circles representing strong experimental support. **B** Representative diagram of the effect that titin modifications have on the passive force development during sarcomere stretch. **C** Structure of Lim domains ½-1 of FHL2. Coordination of Zinc ions into specialized Zinc-fingers by side-chains in FHL2 is highlighted in the representation. Structure adapted from RCSB accession 2MIU. **D** Characterized FHL1 splice isoforms and domain layout. NLS nuclear localization signal, NES nuclear export signal. Figure adapted from Domenighetti et al. (2014)

Another flexible linker within the I-band of titin is the N2B region (Linke et al. 1999). This region is found in all cardiac titin isoforms: the ‘smaller’ 2.97 MDa N2B and the larger 3.3 MDa N2BA titin splice variants (Freiburg et al. 2000). Similar to N2A, the N2B region is located N-terminal to titin’s PEVK element, but in closer proximity to the Z-disc (Freiburg et al. 2000; Gautel et al. 1996; Labeit and Kolmerer 1995; Linke et al. 1999). Flanking the N2B region are Ig-domains I23 and I26, with Ig-domains I24 and I25 separating three individual N2B unique sequences (Figure 3A). C-terminal of the tandem arrangement of I23-N2B_us1_-I24-N2B_us2_-I25-N2B_us3_-I26 is the constitutive I27 domain, which marks the beginning of a heavily varied Ig-domain segment in titin that originates from alternative splicing and exon skipping events (Freiburg et al. 2000).

Expression of the N2B region in cardiac cells has been shown to disrupt thin-filament structure, while a similar experiment using the N2A region did not show this effect. Further investigation revealed that the Ig-domains N-terminal to the N2B region mediate the disruption of actin filaments, while leaving thick filaments unaffected (Linke et al. 1999). Analysis of binding between N2B_us3_ and actin revealed no direct association of this region in titin to the thin filaments (Yamasaki et al. 2001).

The N2B region contains sites for several types of posttranslational modifications that are able to modulate passive force development and titin stiffness (Figure 3B) (Borbely et al. 2009; Fukuda et al. 2005; Hamdani et al. 2013a; Lee et al. 2010; Nedrud et al. 2011). Among the protein kinases shown to modify this region of titin are cAMP-dependent protein kinase (PKA) (Fukuda et al. 2005; Kruger and Linke 2006; Yamasaki et al. 2002), extracellular signal-regulated kinase-2 (Erk2) (Raskin et al. 2012), calcium/calmodulin-dependent kinase 2 (CaMKII) (Hamdani et al. 2013b) and cGMP-dependent protein kinase (PKG) (Kruger et al. 2009). Intriguingly, one mapped phosphorylation site within N2B_us3_, S4139 in NP_003310.4, was targeted by both, PKA and PKG. PKG also modifies proximal Ig-domains I24-I25. Reports indicate that the phosphorylation state of titin at the N2B_us3_ sequence may be modulated by several of its interaction partners (Krysiak et al. 2018; Raskin et al. 2012). Besides phosphorylation, the N2B region may also be subject to cysteine cross-linking caused by prevailing oxidative conditions, such as found in heart failure patients (Beckendorf and Linke 2015; Grieve and Shah 2003; Grutzner et al. 2009). Common among most identified posttranslational modifications in the N2B region is the ability to modulate passive titin mechanics. Phosphorylation of the unique sequence N2B_us3_ resulted in decreased passive stiffness (Fukuda et al. 2005; Kruger et al. 2009; Kruger and Linke 2006; Raskin et al. 2012; Yamasaki et al. 2002), while formation of disulfide bridges by cysteine residues led to increased titin-based stiffness (Grutzner et al. 2009; Nedrud et al. 2011).

The importance of the N2B unique sequence for muscle development and physiology is underscored by pathological mutations (Itoh-Satoh et al. 2002; Matsumoto et al. 2005) and vertebrate knockout models (Nedrud et al. 2011; Radke et al. 2007; Xu et al. 2002). Excision of exon 49 of the mouse titin gene, which leads to an absence of the N2B region, resulted in mice that were viable, albeit displaying smaller heart sizes with diastolic dysfunction (Radke et al. 2007). Notably, the expression levels of some but not all of the known binding partners for titin N2B were affected by the deletion. The phenotype in the N2B knockout model can be ameliorated by reduction of RNA binding motif protein 20 (Rbm20) protein levels (Hinze et al. 2016). Rbm20 is a trans-acting splicing factor highly expressed in striated muscles, especially in the heart (Brauch et al. 2009). Mutations in Rbm20 have been observed in at least 2–3% of familial dilated cardiomyopathy (DCM) cases (Brauch et al. 2009; Kayvanpour et al. 2017; Refaat et al. 2012). While the most frequently investigated target for Rbm20’s modulation of mRNA splicing is titin itself (Guo et al. 2012), over 30 genes involved in striated muscle and muscle fiber homeostasis have been shown to be a target of Rbm20, including the N2B interacting four-and-a-half LIM domain proteins FHL1 and FHL2 (Hinze et al. 2016; Hutchinson et al. 2015; Lennermann et al. 2020).

Mutant zebrafish that have a deletion of the N2B region resulted in a more severe phenotype. Mutants exhibited dilated cardiomyopathy with poor contractility caused by disrupted sarcomerogenesis (Xu et al. 2002). Notably, morpholino guided ablation of the N2B region phenocopied the cardiac phenotype of the mutant zebrafish.

While the N2B region of titin exerts biomechanical effects on cardiac stiffness, it also acts as a signaling hub, by offering binding sites to multiple interaction partners. Binding partners include proteins of the four-and-a-half LIM (FHL) protein family (Krysiak et al. 2018; Lange et al. 2002; Sheikh et al. 2008), the chaperones Hsp90 (Krysiak et al. 2018) and αB-crystallin (Bullard et al. 2004), as well as protein phosphatase 5 (PP5) (Krysiak et al. 2018).

## N2B association with chaperones and modifying enzymes

The chaperones αB-crystallin and Hsp90 are members of the heat shock protein family. αB-crystallin is highly expressed in the lens but can also be found in striated muscles (Bhat and Nagineni 1989), where its localization to myofibrils increases during stress (Golenhofen et al. 1999). Further analysis of the myofibrillar localization revealed that αB-crystallin binds several regions within titin, including N2B_us3_ and the adjacent Ig-domains I26 and I27 (Bullard et al. 2004). The authors speculated that association of αB-crystallin to N2B_us3_ and the adjacent Ig-domains indicates vulnerability of these regions/domains to misfolding under physiological conditions. However, association of the chaperone to titin under normal physiological conditions may also serve as anchoring point to form a reservoir of the rather abundant αB-crystallin, which makes up 3–5% of soluble protein in cardiac muscle (Horwitz 2000). Indeed, the exclusive association of the chaperone with the N2B region became more diffuse and extended towards the Z-disk upon unphysiological stretching of the sarcomeres (Bullard et al. 2004), indicating association of αB-crystallin with unfolded proximal Ig-domains in titin that are known to be less stable (Li et al. 2002; Watanabe et al. 2002). Atomic force microscopy measurements of αB-crystallin action on isolated stretched Ig-domains showed a stabilizing effect in the presence of this chaperone (Bullard et al. 2004). Some of the kinases that phosphorylate N2B_us3_ and adjacent Ig-domains modify also αB-crystallin, which partially correlates with increased myofibrillar localization of the protein (Golenhofen et al. 1999). Binding of Hsp90 to the N2B region may also be mediated by the phosphorylation state of titin, as hypophosphorylated N2B resulted in stronger I-band localization of this chaperone (Krysiak et al. 2018). In addition to N2B, Hsp90 can also associate with Erk2, whose localization to the titin I-band is in turn mediated by FHL proteins (Raskin et al. 2012; Sheikh et al. 2008).

Binding of PP5 to the N2B_us3_ was mapped to the catalytic domain in the phosphatase, with amino acids 205-211 being critical fort the interaction (Krysiak et al. 2018). The binding is increased upon phosphorylation of N2B_us3_, specifically when mediated by PKA. Association of the phosphatase with N2B_us_ led to dephosphorylation of titin, with Erk2-sites proving particularly vulnerable to the phosphatase. Transgenic mice that overexpress PP5 display hypophosphorylation of N2B_us3_ and increased passive tension. PP5 also binds to FHL1, another known binding partner of N2B_us3_, without altering FHL localization.

## FHL proteins and protein kinases as regulators for titin-based signaling and stiffness

The best characterized interaction partners for N2B_us3_ are members of the four-and-a-half LIM domain protein (FHL) family. Three separate protein homologues, FHL1 (Slim1), FHL2 (DRAL, Slim3) and FHL3 (Slim2), were early on characterized to be highly expressed in cross-striated muscles, with FHL1 and FHL2 being enriched in the heart, while FHL1 and FHL3 are more abundant in skeletal muscles (Chu et al. 2000b; Fimia et al. 2000; Lee et al. 1998b; Morgan and Madgwick 1996; Samson et al. 2004). FHL1 exhibits baseline expression levels in many tissues and becomes specifically upregulated in the heart of many cardiomyopathy models, including MHC^403+/^ mice (Christodoulou et al. 2014), MLP knockouts (Chu et al. 2000b), Gαq and Gsα transgenic mice (Gaussin et al. 2003; Sheikh et al. 2008), as well as in cardiomyopathy patients (Christodoulou et al. 2014). Common among all members of the family is the domain layout, consisting of four-and-a-half LIM domains (named after the first proteins identified to exhibit this fold: LIN-11, Isl-1 and MEC-3). The LIM-domain, a modular protein-binding and protein-dimerization interface, is found in multiple proteins with diverse biological functions. The motif is characterized by a tandem-Zinc finger structure (Figure 3C), with the Zn^2+^-ions being coordinated by a mix of cysteine, histidine and/or an aspartic acid residues using the following conserved sequence: Cys-X_2_-Cys-X_16–23_-His-X_2_-Cys-X_2_-Cys-X_2_-Cys-X_16–21_-Cys-X_2_-Cys/His/Asp (Feuerstein et al. 1994; Kadrmas and Beckerle 2004). All of the FHL proteins were shown to homo and/or heterodimerize (Fimia et al. 2000; Gao et al. 2008; Lange et al. 2002; Li et al. 2001).

### FHL1 and its function for skeletal and cardiac muscles

While ubiquitously expressed and interacting with a host of proteins involved in cell structure, signaling and homeostasis, FHL1 is particularly enriched in striated muscles where it plays an important role in scaffolding or bridging cytoskeletal and cell signaling complexes, and helps regulate gene transcription (Lukash et al. 2020; Shathasivam et al. 2010; Wei and Zhang 2020). FHL family members can exhibit extensive splicing and may display splice isoform-dependent functional diversity. The three major splice isoforms in FHL1 count among the best characterized: FHL1A (also known as Slim1, KyoT1), FHL1B (Slimmer, KyoT3 or transcript variant 1) and FHL1C (KyoT2 or transcript variant 4) are all composed of a half LIM followed by four, three and two full LIM domains, respectively (Figure 3D) (Brown et al. 1999; Chu et al. 2000b; Taniguchi et al. 1998). Analysis of alternative start-site detection by 5′RNA-seq using RNA from cardiomyopathy patients and rodent models revealed further that FHL1A exists in two variants, a transcript that is expressed basally in healthy hearts (transcript 3, bFHL1), and a slightly larger version of FHL1A (transcript 2, iFHL1) that bears 48 additional nucleotides or 16 extra amino acids at the N terminus of the protein, whose expression was highly induced in all investigated cardiomyopathy types (Christodoulou et al. 2014; Domenighetti et al. 2014). This alternate FHL1A is the most abundant isoform expressed in skeletal muscles, where it is suggested to play an essential role in myoblast fusion, muscle fiber formation, myofibrillar organization and contractile function (Cowling et al. 2008; Domenighetti et al. 2014; Loughna et al. 2000; Robinson et al. 2003). Differential splicing of the FHL1 gene (eight exons, located on the X chromosome in both humans and rodents) also leads to the presence of a nuclear-addressing sequence and/or RPB-J-binding domain in the C terminus of the FHL1B and FHL1C isoforms, promoting their nuclear localization. While predominantly sarcomeric (e.g. Z-disk in skeletal muscles, I-band in the heart), FHL1 isoforms have also been shown localize to the sarcolemma and mitochondria in mature skeletal muscle fibers, to focal adhesions in myoblasts and the nucleus in satellite cells (Cottle et al. 2009; Domenighetti et al. 2014; McGrath et al. 2006). The sarcomeric localizations may be dependent on the presence/absence of the cardiac specific titin N2B splice isoform (Blandin et al. 2013; Raskin et al. 2012; Sheikh et al. 2008), or FHL1 interaction with other sarcomeric proteins, such as myosin-binding protein C (Blandin et al. 2013; McGrath et al. 2006) or MLP (Csrp3) (Blandin et al. 2013).

Over the past 10+ years, FHL1 has been identified as the causative gene mutated in at least six distinct myopathies affecting skeletal and cardiac muscles with a widely variable clinical manifestation in affected patients. The most observed are reducing body myopathy (RBM), Emery-Dreifuss muscular dystrophy (EDMD), X-linked myopathy characterized by postural muscle atrophy (XMPMA) and scapuloperoneal myopathy (SPM) (reviewed in Cowling et al. (2011)). RBM is the most severe of the FHL1-linked myopathies. Various sporadic or inherited missense mutations that most commonly disrupt the 2nd LIM domain of FHL1 induce the formation of aggresome-like cell inclusions in RBM. These aggresomes incorporate both mutant and wild-type FHL1, as well as other proteins (e.g. desmin, ubiquitin, actin, dystrophin, myosin heavy chains) trapped in a gain-of-function and dominant-negative manner. The age of onset ranges from infancy to childhood, and in some cases adult onset is observed. At the severe end of the spectrum, early onset sporadic cases of RBM often present with motor delay in the first years of life, with rapid decline of skeletal muscle function leading to loss of ambulation before puberty and progression to respiratory failure and death. Cardiac involvement is less common in RBM, but dilated cardiomyopathy and heart failure have been observed in severe cases (Schessl et al. 2010; Schessl et al. 2011; Schreckenbach et al. 2013). A cardiac phenotype often manifests as hypertrophic or dilated cardiomyopathy and is the most serious symptom in FHL1-induced EDMD patients. It can precede joint contractures and muscle weakness and lead to progressive abnormalities in the cardiac conduction system resulting in heart block and sudden death (Puckelwartz and McNally 2011). FHL1 gene mutations identified in EDMD patients include point mutations, insertions and deletions that lead to reduced FHL1 levels in most patients. This finding suggests that loss of normal protein function via reduced FHL1 protein expression and impaired binding of interaction partners may be important in disease pathogenesis. Most of the EDMD-linked mutations in FHL1 are located in the most distal exons (5–8) of the gene and differentially affect expression and splicing of the three main FHL1 isoforms (Gueneau et al. 2009; Tiffin et al. 2013; Ziat et al. 2016). Collectively, these clinical studies define mutations disrupting LIM domains in FHL1 proteins as a novel, but important cause of human striated muscle disease. In addition, these mutations are commonly associated with a widely variable clinical presentation in affected patients. This variability may relate to the multifaceted roles of FHL1 isoforms in striated muscles, including their ability to function as molecular adaptor and scaffolding proteins within the sarcomere in conjunction with their regulation of cell signaling within the cytosol and gene transcription within the nucleus.

In physiological conditions, baseline FHL1 levels are significantly lower in the heart when compared to skeletal muscles (Chu et al. 2000b; Domenighetti et al. 2014). However, FHL1 expression is increased in idiopathic pulmonary arterial hypertension patients, in patients with LV hypertrophy and hypertrophic cardiomyopathy (Hwang et al. 1989; Lim et al. 2001), as well as in mice with agonist, pressure or volume-overload cardiac hypertrophy (Chu et al. 2000b; Gaussin et al. 2003; Hutchinson et al. 2015). FHL1 is also upregulated in mouse models of cardiomyopathy induced by myosin heavy chain missense mutations (MHC^403/+^) (Christodoulou et al. 2014), MLP deletion (Chu et al. 2000b), in Gαq and Gsα transgenic mice (Gaussin et al. 2003; Sheikh et al. 2008) as well as mice that overexpress β-adrenergic receptors (Gaussin et al. 2003). Elevated FHL1-levels in Gsα transgenic mice are reduced by pharmacological inhibition of β-adrenergic receptors (Gaussin et al. 2003), suggesting activation of G-protein coupled receptor (GPCR) signaling as one of the main drivers for FHL1 expression. FHL1 knockout mice provided intriguing mechanistic insights into FHL1 expression in hearts. Loss of FHL1 in Gαq transgenic mice has been demonstrated to rescue the cardiomyopathy phenotype (Sheikh et al. 2008). However, in α-myosin heavy chain R403Q missense mutation mice (MHC^403/+^), another mouse model for hypertrophic cardiomyopathy with elevated levels of FHL1, ablation of the gene had opposing effects and exacerbated the cardiomyopathy phenotype (Christodoulou et al. 2014). Intriguingly, FHL1 may also modulate GPCR signaling by binding to Pleckstrin homology and RhoGEF domain containing G2 (Plekhg2) (Sato et al. 2016), a Rho family-specific guanine nucleotide exchange factor that is known to associate with small G-proteins Gβɣ and Gsα (Sugiyama et al. 2017). While Plekhg2 is known to be expressed in heart or skeletal muscles (Nishikawa et al. 2021), its role in modulating FHL1-dependent signaling in cross-striated muscles has not been further investigated. The linkage of FHL1 gene activity to GPCR signaling suggests a positive feedback loop that may be modulated by actions of FHL1 itself. Indeed, it has been demonstrated that myocardial overexpression of FHL1 could be induced by transcription of the FHL1A isoform encoded by the alternate upstream start-site (Christodoulou et al. 2014) and/or through modulation of FHL1B levels (Hinze et al. 2016).

**Fig. 4.**
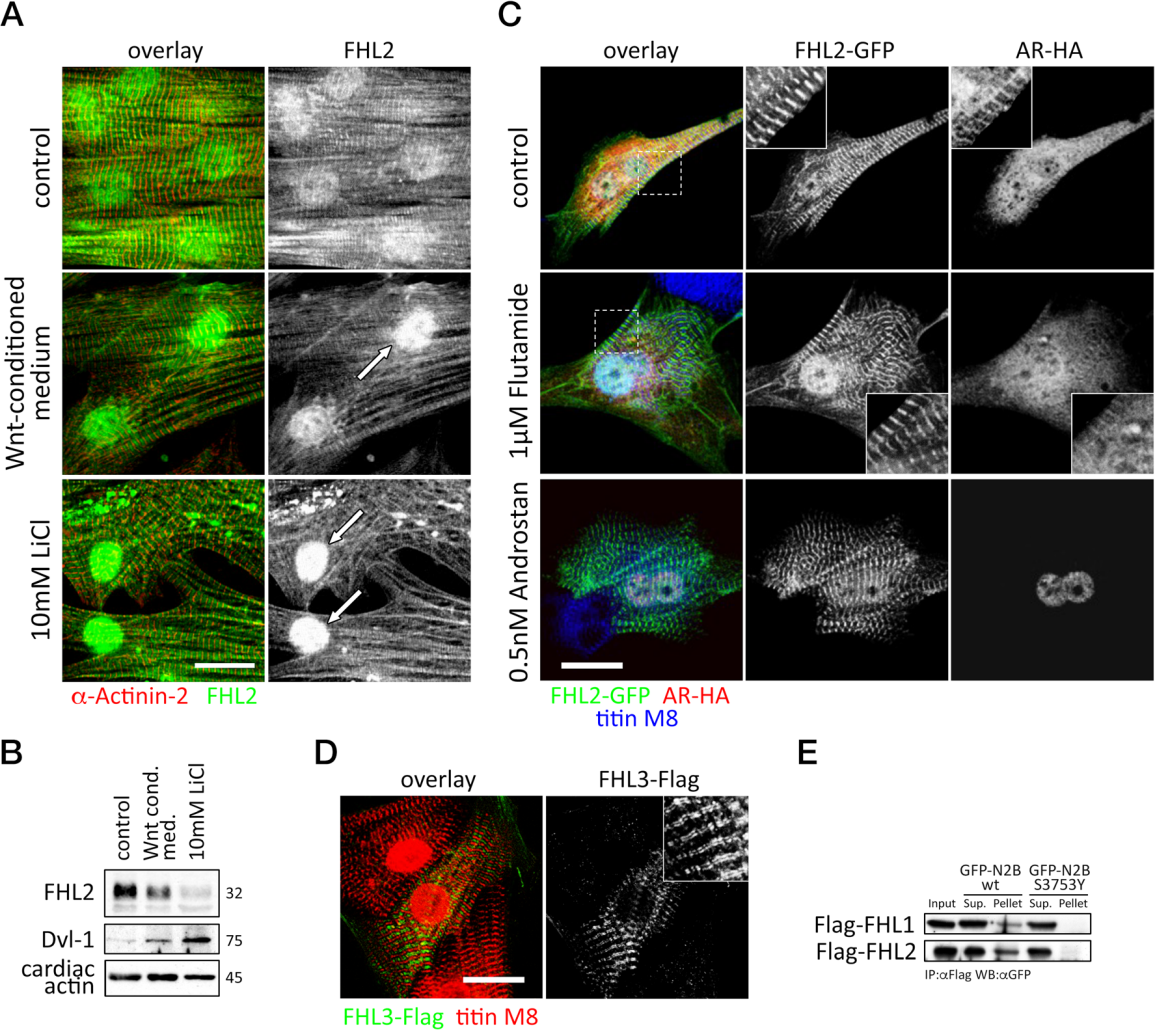
**A–B** Localization (**A**) and expression levels (**B**) of endogenous FHL2 (green in the overlay in (A)) in neonatal rodent cardiomyocyte cultures treated with Wnt-conditioned medium, 10 mM LiCl or vehicle treated controls. Arrows in (**A**) indicate increased nuclear localization of FHL2. Sarcomeric counterstain in (**A**) was sarcomeric α-actinin (red in the overlay), while cardiac actin served as loading control in (**B**). Dvl-1 expression levels indicated activation of Wnt signaling. Scalebar = 10 μm. **C** Analysis of HA-tagged androgen receptor (AR, red in the overlay) and GFP-tagged FHL2 localization (green in the overlay) in transfected rat cardiomyocyte cultures, treated with 0.5 nM of AR agonist androstan, 1 μM AR antagonist flutamide or vehicle controls. Titin-M8 served as sarcomeric counterstain (blue in the overlay). Scalebar = 10 μm. **D** Analysis of Flag-tagged FHL3 localization (green in the overlay) in transfected neonatal rodent cardiomyocyte cultures. Titin-M8 served as sarcomeric counterstain (red in the overlay). Scalebar = 10 μm. **E** Analysis of wildtype and SY-mutant titin N2B binding to FHL1 or FHL2 in co-immunoprecipitation assays shows loss of FHL interaction with mutant titin. Sup supernatant. All figures were adapted from Lange (2005) with permission of the author

Since its discovery in 1998 (Lee et al. 1998a), FHL1 has emerged as an important regulator of passive/diastolic tension in stress-induced left ventricular (LV) cardiac hypertrophy. In particular, studies performed in FHL1 knockout mice have shown that FHL1 is part of a biomechanical stress sensor complex that scaffolds mitogen-activated protein kinase (Mapk) components (Erk2, Raf1, Mek2) to the sarcomeric titin N2B_us3_ spring element, regulating diastolic function and cardiac stress responses through N2B_us3_ phosphorylation and Mapk signaling (Raskin et al. 2012; Sheikh et al. 2008). A recent study demonstrated that protein phosphatase 5 (PP5) is also a key element of this N2B_us3_ and FHL1 stress-sensing signalosome, modulating muscle compliance by dephosphorylating the N2B_us3_ region (Krysiak et al. 2018). A human mutation in titin (N2B_us3_ S3799Y) that affects FHL2 binding also significantly impacted Mapk/Erk2-mediated titin N2B_us3_ phosphorylation (Itoh-Satoh et al. 2002; Matsumoto et al. 2005; Raskin et al. 2012). Our data indicate that the N2B_us3_ S3799Y mutation similarly affects FHL1 association with titin (Figure 4E) (Lange 2005). Summarily, these studies provide clinical relevance for the function of the FHL1/PP5/Mapk-containing signalosome for N2B_us3_ phosphorylation and the development of human cardiomyopathy.

FHL1 was also found to be upregulated in a titin PEVK knockout mouse model that developed cardiac hypertrophy and increased passive tension (Granzier et al. 2009), strengthening the importance for an interaction between titin’s molecular spring elements and FHL1 during regulation of LV passive/diastolic tension. Intriguingly, these FHL1-dependent mechanisms, which have been extensively investigated in the left ventricle (LV), may not be involved in stress-induced maladaptive remodeling of the right ventricle (RV) (Veith et al. 2020). The reasons for these differences between the RV and LV remain unclear.

### FHL2 and its role in the heart

FHL2 was initially identified as downregulated gene in a rhabdosarcoma cell line but was subsequently found to be highly expressed in the heart where it exhibits a sarcomeric localization (Chan et al. 1998; Genini et al. 1997; Scholl et al. 2000). Later studies revealed two binding sites for FHL2 within titin: a predominant interaction site within the N2B_us3_ region of I-band titin, and another weaker association to the is2 sequence located at the sarcomeric M-band (Lange et al. 2002). Pathological mutations in the N2B_us3_ region of titin that have been associated with the development of hypertrophic cardiomyopathy were shown to affect binding of FHL2 (Itoh-Satoh et al. 2002; Matsumoto et al. 2005) and FHL1 (Figure 4E). Besides titin, FHL2 was shown to interact with other sarcomeric proteins, such as slow myosin binding protein-C (MyBP-C) (McGrath et al. 2006), the thin filament capping protein (Huttlin et al. 2015), and components of the cardiac intercalated disc, such as plakoglobin (Luck et al. 2020; Rolland et al. 2014) and β-catenin (Wei et al. 2003). Pathway enrichment analysis for the over 200 known binding partners for FHL2 catalogued in the protein interaction database BioGrid (Stark et al. 2006) suggests roles for FHL2 in cancer (diseases in signal transduction and second messengers); Smad, Notch and Wnt signaling; and functions for the protein in regulating transcription factor activity that are also supported by its shuttling to the nucleus in many cell-types (Genini et al. 1997; Huang et al. 2019; Labalette et al. 2004; Scholl et al. 2000; Wei et al. 2003). Shuttling of FHL2 between its sarcomeric localization and the nucleus as well as FHL2 protein levels may be modulated by some of these cellular signaling pathways. An example is the canonical Wnt/β-catenin signaling pathway, which altered FHL2 localization and levels in neonatal mouse cardiomyocyte cultures treated with Wnt-conditioned medium or 10 mM LiCl (Figure 4A–B) (Lange 2005). A possible link between FHL2 and β-catenin signaling in the heart may also be supported by poor postnatal viability of FHL2/β-catenin double knockouts (Table 1, data kindly provided by Alain Hirschy, Evelyne Perriard and Jean-Claude Perriard (Hirschy 2005)), compared to cardiac specific β-catenin knockouts (Hirschy et al. 2010) or FHL2 knockout mice alone (Chu et al. 2000a).

**Table 1:** Mendelian ratios of conditional β-catenin and FHL2 global knockout crossings. Table adapted from Hirschy (2005) with permission of the author

	Crossing of conditional cardiac specific β-catenin knockouts (Hirschy et al. 2010) in a global FHL2 knockout background (Chu et al. 2000a). Please note, only cre-positive wildtype and double knockout animals are counted.			
Age	P0		P21	
Genotype	FHL2 knockout β-catenin wildtype	FHL2 knockout β-catenin knockout	FHL2 knockout β-catenin wildtype	FHL2 knockout β-catenin knockout
Expected ratios	50%	50%	50%	50%
Number of animals and %	25 (52%)	23 (48%)	24 (71%)	7 (29%)

For the heart, FHL2 is thought to serve as a linker protein that was shown to mediate the binding of metabolic enzymes (Lange et al. 2002) and extracellular signal-regulated kinase 2 (Erk2) to the I-band of the sarcomere (Purcell et al. 2004). Intriguingly, only activated Erk2 is bound by FHL2, which in addition prevents translocation of the kinase to the nucleus and activation of its transcriptional activity. FHL2 may also serve as a sarcomeric anchor for other binding partners involved in cellular signaling pathways. One such example is the androgen receptor, whose binding to FHL2 and role as tissue-specific co-activator have been known for some time (Muller et al. 2000). Analyzing the subcellular localizations of HA-tagged androgen receptor and GFP-tagged FHL2 in transfected neonatal rat cardiomyocytes cells revealed sarcomeric colocalization of both proteins in untreated and antagonist treated cells (Figure 4C) (Lange 2005). Activation of the androgen receptor by the agonist androstan resulted in shuttling of androgen receptor to the nucleus. Of note: some nuclear staining of FHL2 was present in all treatment conditions, suggesting two pools of FHL2, a sarcomere-bound fraction that could serve as anchor for multiple binding partners, and a nuclear pool that may assist in transcriptional gene-regulation. Indeed, overexpression of FHL2 in isolated cardiomyocytes was shown to reduce cardiac hypertrophy due to Mek1, Gata4 and phenylephrine stimulation (Purcell et al. 2004). Nevertheless, while FHL2 knockouts displayed greater cardiac hypertrophy following catecholamine stimulation (Kong et al. 2001), global loss of the protein is well tolerated, and the hypertrophic response following transverse-aortic constriction (TAC) was indistinguishable from that of controls (Chu et al. 2000a).

### Enigmatic roles of FHL3

Despite its high expression levels in skeletal muscle (Lee et al. 1998b), FHL3 functions remain more enigmatic compared to FHL1 and FHL2. FHL3 mRNA was found to be upregulated during skeletal muscle differentiation and regeneration (Han et al. 2019; Meeson et al. 2007), suggesting roles for this FHL protein in muscle formation. Moreover, mRNA levels of FHL3 were found to be reciprocally regulated to that of FHL1, the other FHL family member expressed in skeletal muscles: FHL3 expression peaked early during differentiation, while FHL1 displayed increased expression in more mature myotubes (Morgan and Madgwick 1999). In undifferentiated myoblasts, FHL3 localizes to the nucleus and actin stress fibers, partially due to its binding to actin filaments (Coghill et al. 2003; Sun et al. 2020). This localization changes in mature muscles, where FHL3 is found at the Z-disk, presumably by binding to sarcomeric α-actinin (Coghill et al. 2003). Analysis of FHL3 roles during differentiation indicated further that the protein regulates myosin heavy chain isoform expression by modulating MyoD and pCreb transcriptional activity (Cottle et al. 2007; Zhang et al. 2016). Similar to other FHL proteins, FHL3 interacts with Hif1α and modulates this hypoxic signaling pathway (Lin et al. 2012).

For the heart, FHL3 was found to be significantly deregulated in ischemic and dilated cardiomyopathy (Molina-Navarro et al. 2014). However, the relevance of this finding is unclear as the protein is generally thought to be extremely low in the heart (Lee et al. 1998b). Nevertheless, FHL3 also localizes to the sarcomeric I-band in transfected cardiomyocytes, suggesting either direct binding to titin N2B_us3_ or indirect association to titin through heterodimer formation with FHL1 and/or FHL2 (Figure 4D) (Lange 2005). To date, in vivo functions for FHL3 have not been further explored, as no knockout or transgenic animals are available.

### Functional redundancy of FHL protein action?

The lack of a baseline cardiac phenotype particularly in FHL2 knockout mice (Chu et al. 2000a), but also in FHL1 knockouts (Sheikh et al. 2008) is surprising given the number of binding partners and their implied significance for the hypertrophic response of the heart. However, functional redundancy between FHL1 and FHL2 may play a more profound role than previously anticipated. Common among known binding partners for FHL1, FHL2 and/or FHL3 are titin (Lange et al. 2002; Matsumoto et al. 2005; Sheikh et al. 2008), MyBP-C (McGrath et al. 2006), actin and sarcomeric α-actinin (Coghill et al. 2003; Sun et al. 2020), as well as several key proteins involved in cellular signaling pathways, such as Hif1α (Hubbi et al. 2012; Lin et al. 2012), α- and β-subunits of the integrin receptor (Samson et al. 2004), Erk2 (Purcell et al. 2004), Smad2/3/4 (Ding et al. 2009) or the serum response factor (Srf) (Philippar et al. 2004). Both, FHL1 and FHL2 expression and function are also known to be linked to β-adrenergic GPCR signaling in hearts (Gaussin et al. 2003; Kong et al. 2001; Sato et al. 2016; Sheikh et al. 2008). In addition, FHL proteins are known to form homo- and heterodimers (Fimia et al. 2000; Gao et al. 2008; Lange et al. 2002; Li et al. 2001), suggesting combinatorial functionality of their biological roles that may depend on the expression level of each of the FHL protein at any given time in cardiac and skeletal muscles. Intriguingly, the redundancy may also be reflected in the finding that loss of FHL1 or FHL2 in the heart does not result in a measurable cardiac phenotype at baseline. FHL1 was also not upregulated upon loss of FHL2 at baseline (Chu et al. 2000a); however, it becomes the predominant FHL protein induced by TAC and in a model of dilated cardiomyopathy (Chu et al. 2000b). In contrast to the heart, loss of FHL1 in the skeletal muscles, where no expression of FHL2 on the protein level can be detected in slow and fast-twitch muscles (Kong et al. 2001; Lange et al. 2002), was associated with the development of myophaties in both, mice and humans. While skeletal muscle also express FHL3, their differential developmental expression profile (Morgan and Madgwick 1999) may contribute to a lack of functional redundancy that compensates for the loss of FHL1. Ultimately generation of double and/or triple knockout models may shed some light into functional overlap and redundance between FHL proteins. Mendelian ratios of global knockout mice at weaning suggest that FHL1/FHL2 double knockouts are lost during development (Table 2). However, exact timepoints and reasons for the lack of double knockouts remain to be discovered.

**Table 2:** Mendelian ratios of FHL1 and/or FHL2 global knockout crossings

	FHL1. FHL1 global knockout (Sheikh et al. 2008). Breeding of wildtype males with heterozygous FHL1 females. Please note, FHL1 is X-linked.		
Genotype	Wildtype (both males and females)	Heterozygous (females only)	Hemizygous (males only)
Expected ratios	50%	25%	25%
Number of animals and percent of genotyped animals at weaning stage (P21)	91 (55.5%)	35 (21.3%)	38 (23.2%)
	FHL2. FHL2 global knockout (Chu et al. 2000a). Crossing of heterozygous males and females.		
Genotype	Wildtype	Heterozygous	Homozygous
Expected ratios	25%	50%	25%
Number of animals and percent of genotyped animals at weaning stage (P21)	26 (22.6%)	60 (52.2%)	29 (25.2%)
	FHL1/2 double knockouts. Crossing of FHL1 (y/+); FHL2 (−/−) males with FHL1 (+/−); FHL2 (−/−) females. Please note that the breeding was performed using a FHL2 homozygous knockout background that showed no changes to viability or fertility (Chu et al. 2000a).		
Genotype	FHL2 knockout, FHL1 Wildtype (both males and females)	FHL2 knockout , FHL1 Heterozygous (females only)	FHL2 knockout , FHL1 Hemizygous (males only)
Expected ratios	50%	25%	25%
Number of animals and percent of genotyped animals at weaning stage (P21)	29 (69.1%)	9 (21.4%)	4 (9.5%)

Besides serving as a sarcomeric anchor for Mapk signaling-associated kinases, FHL1 was also characterized to modulate phosphorylation sites within N2B_us3_ (Raskin et al. 2012), potentially altering titin-based cardiac stiffness. Presence of FHL1 in Erk2 kinase assays resulted in significantly reduced N2B phosphorylation by Erk2, suggesting blockage of access to the serine/threonine residues in titin. This finding is contested as FHL1 knockout hearts display reduced phosphorylation of titin at S3991 compared to wildtype hearts (Krysiak et al. 2018). However, Krysiak et al. have not taken into consideration effects of FHL2 when interpreting their results. While wildtype hearts have both FHL proteins present that compete for binding to titin, loss of FHL1 results in the exclusive association of FHL2 to the N2B_us3_ element in titin. Binding of FHL2 to titin and/or Erk2 may account for the altered titin phosphorylation observed in FHL1 knockout hearts, although it remains to be determined if FHL2 plays similar roles in blocking access to the same phosphorylation site(s) as FHL1. Another interpretation of the results presented by Krysiak and coauthors could be that FHL2 blocks titin phosphorylation at S3991 more efficiently than FHL1. Further experimental evidence is needed to fully understand the role that FHL proteins exert on Erk2-based titin phosphorylation and the resulting change in cardiac stiffness.

Intriguingly, FHL proteins may not only modulate passive muscle mechanics, but could also function as mechanosensors in many cell types, including cross-striated muscles. A recent manuscript by Sun and co-authors indicated that while all FHL proteins associate with thin filaments, binding of FHL2 and FHL3 (and to a lesser extend FHL1) to F-actin is greatly enhanced in the presence of mechanical force (Sun et al. 2020). While this mechanism may play a role in controlling the nuclear shuttling and co-transcriptional activity of FHL proteins in non-muscle cells (Nakazawa et al. 2016; Sun et al. 2020), it remains to be demonstrated for cross-striated muscles.

Another function of FHL proteins that may be somewhat conserved between FHL1 and FHL2 is their association with metabolic enzymes. FHL2 has been suggested to tether phosphofructokinase, adenylate kinase and muscle creatine kinase to the sarcomeric I-band in cardiomyocytes (Lange et al. 2002), and to bind to pyruvate dehydrogenase (Pdhb) (Huttlin et al. 2015). Loss of FHL2 in mice leads to reduced weight gain by diet-induced obesity, higher energy expenditure, browning of white adipose tissue and enhanced glucose uptake and consumption of the heart (Clemente-Olivo et al. 2021). Analysis of mRNA expression in the heart suggests significant modulation of the X nuclear receptor (Lxr)/retinoid X receptor (Rxr), Mapk and Erk signaling and glucose metabolism pathways.

FHL1 was shown to interact with Eno1, another enzyme of the glycolysis pathway (Blandin et al. 2013). The protein also localizes to mitochondria in skeletal muscle, and its loss resulted in morphological abnormalities (Domenighetti et al. 2014).

Redundancy between FHL family members may however also be limited to certain biological roles of these proteins. While seldomly investigated, some studies find differential binding of FHL proteins to interaction partners. A prominent example is the modulation of GPCR signaling by FHL1A and FHL1B via their interaction partner Plekhg2, which does not extend to FHL1C and other members of the FHL protein family (Nishikawa et al. 2019). Another example is FHL2 binding to sphingosine kinase-1, which is not shared by FHL1 or FHL3 (Sun et al. 2006).

Additional studies are required to fully evaluate communality and specificity in FHL protein function.

## Beyond the N2A and N2B regions: the titin PEVK element and the A/I junction

### The elastic PEVK region

Titin’s PEVK-element is encoded by 114 of titin’s 363 exons (Bang et al. 2001a). This region in titin functions as a large, disordered, entropic spring (Linke et al. 2002). Splicing of titin and length regulation of the PEVK element is under control of the RNA splice factor Rbm20 (Li et al. 2013; Li et al. 2012; Methawasin et al. 2014). The PEVK element consists of two repeating motifs called PPAK (proline, proline, alanine, lysine) and PolyE (Glutamic acid), encompassing ~75% and ~25% of this region in titin, respectively (Duan et al. 2006). The PPAK motif is formed by short 26–28 positively charged amino acid repeats that are estimated to fold mostly into α-helices and disordered regions (Duan et al. 2006). PolyE motifs consist of ~45% glutamic acid, providing a negative charge to the PPAK repeats, and are considered fully disordered in structure (Duan et al. 2006). Some secondary structure has been ascribed to the PEVK region, but current studies disagree on the stability of said structure in vivo (Gutierrez-Cruz et al. 2001; Ma and Wang 2002; Ma and Wang 2003). The alternating arrangement of the positively charged PPAK and negatively charged PolyE motifs is thought to contribute to the elastic nature of the PEVK region (Greaser 2001; Huber et al. 2012; Linke et al. 1998; Yamasaki et al. 2001).

#### PEVK interacting proteins

PEVK interactions with F-actin, partly mediated through calcium, form another mechanism for increasing passive tension (Figure 3A) (Bianco et al. 2007; Chung et al. 2011; Nagy et al. 2004). The PEVK region can also be phosphorylated by PKCα (Hidalgo et al. 2009) and calcium/calmodulin-dependent kinase 2 (CaMK-IIδ) (Hamdani et al. 2013b; Hidalgo et al. 2013) at S11878 and S12022 (N2B isoform, SwissProt: Q8WZ42). Phosphorylation at these serine residues increases stiffness of the PEVK element (Figure 3B) (Anderson et al. 2010; Hamdani et al. 2013b; Hidalgo et al. 2009; Hidalgo et al. 2013), which can be reversed by protein phosphatase 1 (PP1) (Hidalgo et al. 2009).

Beyond actin, there are few proteins whose binding has been mapped to the PEVK element. The S100-family of proteins known for binding calcium (Donato et al. 2013) interacts with titin PEVK (Yamasaki et al. 2001). S100A1 seems to inhibit the interaction between actin and the PEVK region (Yamasaki et al. 2001) and may provide a mechanism for modulating titin stiffness. Additionally, S100A1 can disrupt the interaction of nebulin’s SH3-domain with the PEVK region (Gutierrez-Cruz et al. 2001). The role of nebulin-PEVK interaction is unknown, but it has been suggested to aid nebulin localization to the thin filament (Ma and Wang 2002). Lastly, calpain 3 can bind in the PEVK region where it is thought to form a reservoir for proteolytic cleavage of titin (Ojima et al. 2014). Signaling in the PEVK region is not very well understood, but the recent publication of two PEVK deletion models: TtnΔEx112-158 (Brynnel et al. 2018) and TtnΔEx219-225﻿ (Granzier et al. 2009; van der Pijl et al. 2020), showed hypertrophic remodeling in skeletal muscles. Whether these models represent propagation of mechanosignaling from the N2A and/or N2B regions or untapped mechanosensing hotspots in the PEVK region remains to be studied.

### Titin’s A/I junction

The A/I junction of titin (Figure 1D) is structurally similar to both the D-zone and C-zone of A-band titin. Although it comprises Ig- and Fn3-domains, this region of titin does not follow the Ig-(Fn3)_2_-Ig-(Fn3)_3_ repeat of the D-zone or the Ig-(Fn3)_2_-Ig-(Fn3)_3_-Ig-(Fn3)_3_ repeats of the C-zone (Labeit and Kolmerer 1995), and importantly, it does not bind to the thick (myosin) filaments (Bennett and Gautel 1996; Granzier et al. 2014; Muhle-Goll et al. 2001) or myosin binding protein C (MyBP-C) (Bennett et al. 1986; Freiburg and Gautel 1996; Luther et al. 2008). The A/I junction has been proposed to form a stop signal for thick filament polymerization, thereby regulating its length (Bennett and Gautel 1996; Whiting et al. 1989). However, a titin deletion model (TtnΔIAjxn) for the A/I junction showed that thick filament lengths were unaffected. This suggested that myosin filament length regulation may not be directly controlled by this region in titin (Granzier et al. 2014). On the other hand, regulation in thick filament lengths and MyBP-C localization was found in a C1/2-zone deletion mouse model (Tonino et al. 2019; Tonino et al. 2017). This mouse model showed that thick filaments shortened (Tonino et al. 2017) and number of C-stripes decreased (Tonino et al. 2019) when the C1 and C2 modules were deleted from titin. An alternative hypothesis for the function of the A/I junction proposes that this region forms a dimerization sequence for titin (Al-Khayat et al. 2013). It was shown that tryptic fragments of titin’s distal Ig-repeat segment and A/I junction form oligomers (Houmeida et al. 2008). This supports packing of titin into a single multimeric titin filament before fusing with the thick filament, possibly facilitating the organization of the thick filament into the D-zone and C-zone. This hypothesis would also provide a function for the developmental titin ‘cronos’ isoform, with its promoter region in the distal Ig-repeat, which encodes the A-band and M-band regions of titin (Bull et al. 2016; Zaunbrecher et al. 2019; Zou et al. 2015). Thus, the A/I junction does not appear to directly contribute to titin mechanosensing but may perform more structural roles. Studies on the TtnΔIAjxn mouse model indicate that the passive stiffness of the I-band region of titin is important in the development of diastolic dysfunction (Slater et al. 2017) by increasing the extension of the PEVK and N2B regions during sarcomere stretch (Granzier et al. 2014). Interestingly, the N2B interacting protein FHL2 was upregulated in the TtnΔIAjxn mouse (Granzier et al. 2014), supporting that the increased stiffness activates titin mechanosensing in the I-band.

## The emerging therapeutic potential of modulating titin I-band splicing and phosphorylation as well as titin-associated proteins

Changing titin stiffness in disease was shown to alter both N2A and N2B-based signaling (Granzier et al. 2009; Guo et al. 2013; Guo et al. 2018; van der Pijl et al. 2018). Hence, the modulation of titin binding partners as well as titin phosphorylation and isoform ratios are attractive targets for novel therapeutic strategies in the treatment of various cardiomyopathies.

Rbm20, a master regulator for titin I-band splicing, has been shown to provide relief from diastolic heart failure in genetic mouse models (Bull et al. 2016; Hinze et al. 2016; Methawasin et al. 2014). Rbm20 was also able to reverse a heart failure with preserved ejection fraction (HFpEF) like model in mice induced by transverse aortic constriction (TAC) in combination with deoxycorticosterone acetate (DOCA) treatment (Bull et al. 2016). Rbm20 deletion also seemed to rescue mechanically ventilated rats from developing diaphragm weakness (Lindqvist et al. 2018). However, modulation of Rbm20 levels or activity may not be the ‘silver bullet’ for diseases whose underlying pathology can be alleviated by titin length regulation, as the splice factor also processes mRNAs of other muscle genes, such as the ryanodine receptor (RyR), myomesin, nexilin, obscurin or troponin (Maatz et al. 2014). Newer strategies that aim to alter Rbm20 phosphorylation (Murayama et al. 2018) or search for small molecules that selectively modulate Rbm20 targeting (Liss et al. 2018) could decrease unintended off-target effects.

Another promising approach to reverse heart failure in TAC-DOCA mice has been the use of metformin (Slater et al. 2019). This small molecule drug is widely used to treat diabetes by regulating glucose metabolism via AMP-activated protein kinase (AMPK)-dependent and AMPK-independent mechanisms (reviewed in Rena et al. (2017)). Metformin administration in mice improved diastolic dysfunction, mainly by increasing PKA phosphorylation at the N2B region of titin (Slater et al. 2019).

Several studies and a clinical trial investigated the use of phosphodiesterase inhibitor sildenafil to treat diastolic dysfunction in an animal model of HFpEF and in HFpEF patients. Sildenafil inhibits the activity of phosphodiesterase-5 (Pde-5) (reviewed in Andersson (2018)), which increases nitric oxide levels and cGMP-PKG signaling, ultimately resulting in increased phosphorylation at the N2B region of titin (Bishu et al. 2011; Kruger et al. 2009). While the drug showed effectiveness in a dog model of the disease (Bishu et al. 2011), a randomized clinical trial failed to elicit changes to the clinical outcome in patients (Redfield et al. 2013).

An elegant study by Anna-Eliane Hopf and Christian Andresen et al. deciphered changes to titin phosphorylation mediated by the insulin pathway (Hopf et al. 2018). The authors linked increased passive tension in diabetic human and rodent hearts to hyperphosphorylation of the PEVK element (at S11878, UniProtKB: Q8WZ42) and hypophosphorylation of the N2B region (at S4099, UniProtKB: Q8WZ42) in titin. The pathological alterations to kinase activity seen in a rodent model of diabetes could be reversed by treatment with the cardioactive growth-factor neuregulin-1, which successfully increased cGMP-PKG and Erk1/2 activity. The resulting increase in titin phosphorylation at the N2B region (at S4010 via increased Erk1/2 activity, UniProtKB: Q8WZ42) and concomitant decrease of titin PEVK phosphorylation (at S11878 through decreased PKCα activity) lowered passive tension and reduced end diastolic pressures in treated animals.

While altering titin phosphorylation and splicing are established therapeutic routes of great interest, modulating levels and/or localization of titin binding partners of the FHL and Ankrd protein family may emerge as a novel avenue for the treatment of cardiomyopathies. Indeed, reducing Ankrd1 protein levels has been demonstrated to prevent development of dilated cardiomyopathy in mice (Lange et al. 2016). A recent study found that Ankrd1 mRNA levels could be downregulated by inhibiting miR-455-3p (Ueta et al. 2020), a miRNA that was linked to worsened hypertrophic remodeling in mice that underwent pressure overload by transverse aortic constriction (TAC) (Wu et al. 2015). However, it is unclear if the mouse version of miR-455 targets Ankrd1 mRNA, as the sequences show poor evolutionary conservation between mice and humans. Similarly, several studies investigate the modulation of FHL proteins as novel therapeutic strategies, either through the use of miRNA or shRNA that target FHL2 (mostly as a cancer therapy (Brun et al. 2013; Huang et al. 2019)), or through FHL1 overexpression (to treat muscular dystrophy (D'Arcy et al. 2014)). However, experimental animal models that study the loss of FHL proteins indicate positive (Sheikh et al. 2008) and negative effects (Christodoulou et al. 2014; Domenighetti et al. 2014; Kong et al. 2001), largely dependent on the type of cross-striated muscle (cardiac vs. skeletal muscles) or the underlying pathology. Experiments using FHL1 knockout mice crossed into two models for hypertrophic cardiomyopathy serve as an example, whereby the underlying pathology plays a major role on the effect of FHL1 removal. Loss of FHL1 is advantageous in cardiomyopathy caused by Gαq overexpression (Sheikh et al. 2008), but deleterious in the MHC^403/+^ model of hypertrophic cardiomyopathy (Christodoulou et al. 2014). Participation of FHL proteins in several independent hypertrophy pathways may be the primary reason for the divergent outcomes.

To complicate things further, FHL1 is known to display a ubiquitous expression pattern (Fimia et al. 2000), with multiple splice variants expressed under baseline conditions in many tissues at baseline or in disease (Christodoulou et al. 2014; Domenighetti et al. 2014). While targeting of specific splice variants in FHL1 (Christodoulou et al. 2014) may present a possible therapeutic avenue, there is only sparse evidence for the effectiveness of this approach.

Further research will be needed to fully understand the regulation of titin splicing and posttranslational modifications in health and disease, and how to harness small molecules and other interventions for therapeutic purposes in humans. The use of titin-associated proteins as therapeutic targets remains in its infancy, as their biology has not been fully understood.
